# Autophagy Induction by HIV-Tat and Methamphetamine in Primary Midbrain Neuronal Cells of Tree Shrews via the mTOR Signaling and ATG5/ATG7 Pathway

**DOI:** 10.3389/fnins.2018.00921

**Published:** 2018-12-06

**Authors:** Juan Li, Wenguang Wang, Pinfen Tong, Chi-Kwan Leung, Genmeng Yang, Zhen Li, Na Li, Xiaomei Sun, Yuanyuan Han, Caixia Lu, Dexuan Kuang, Jiejie Dai, Xiaofeng Zeng

**Affiliations:** ^1^Center of Tree Shrew Germplasm Resources, Institute of Medical Biology, The Chinese Academy of Medical Science and Peking Union Medical College, Kunming, China; ^2^School of Basic Medicine, Kunming Medical University, Kunming, China; ^3^School of Biomedical Sciences, The Chinese University of Hong Kong, Hong Kong, China; ^4^Chinese University of Hong Kong – Shandong University (CUHK-SDU) Joint Laboratory of Reproductive Genetics, School of Biomedical Sciences, The Chinese University of Hong Kong, Hong Kong, China; ^5^School of Forensic Medicine, Kunming Medical University, Kunming, China

**Keywords:** HIV-Tat, METH, autophagy, dopaminergic neuronal cells, tree shrew

## Abstract

**Background:** Addictive stimulant drugs, such as methamphetamine (METH), increase the risk of exposure to the human immunodeficiency virus-1 (HIV-1) infection and thus predispose individuals to the development of HIV-associated neurocognitive disorders (HANDs). Previous studies have indicated that HIV-Tat (the transactivator of transcription) and METH can synergistically induce autophagy in SH-SY5Y neuroblastoma cells and that autophagy plays a pivotal role in the neuronal dysfunction in HANDs. However, the underlying mechanism of METH-and HIV-Tat-induced neuronal autophagy remains unclear.

**Methods:** We cultured primary midbrain neuronal cells of tree shrews and treated them with METH and HIV-Tat to study the role of METH and HIV-Tat in inducing autophagy. We evaluated the effects of the single or combined treatment of METH and HIV-Tat on the protein expressions of the autophagy-related genes, including Beclin-1 and LC3B, ATG5, and ATG7 in METH and HIV-Tat-induced autophagy. In addition, the presence of autophagosomes in the METH and/or HIV-Tat treatment was revealed using transmission electron microscopy.

**Results:** The results indicated that METH increased the protein levels of LC3B and Beclin-1, and these effects were significantly enhanced by HIV-Tat. Moreover, the results suggested that ATG5 and ATG7 were involved in the METH and HIV-Tat-induced autophagy. In addition, it was found that mTOR inhibition via pharmacological intervention could trigger autophagy and promote METH and HIV-Tat-induced autophagy.

**Discussion:** Overall, this study contributes to the knowledge of the molecular underpinnings of METH and HIV-Tat-induced autophagy in primary midbrain neuronal cells. Our findings may facilitate the development of therapeutic strategies for METH-and HIV-Tat-induced autophagy in HANDs.

## Introduction

Although the clinical implementation of antiretroviral therapy has significantly prolonged the lifetime of the human immunodeficiency virus (HIV-1)-infected population, HIV-associated neurocognitive disorders (HANDs) remain a critical concern in a significant number of HIV-1 positive individuals (Rumbaugh et al., 2008; Maubert et al., 2015). The HIV-1-mediated neuropathogenesis occurs due to the entry of the HIV-1 virus into the central nervous system (CNS) through transmigration of the blood-brain barrier during the early phases of the infection (Zayyad and Spudich, 2015). The neurotoxic activities of numerous HIV proteins, including Gp120, Tat, and Vpr, are also implicated in the neuropathogenic process (Mothobi and Brew, 2012). HIV-Tat (the transactivator of transcription) is a viral protein that compromises the blood-brain barrier permeability (Li et al., 2018) and causes alterations in inflammatory molecule production by interacting with cellular gene promoters and cellular receptors to induce apoptosis, autophagy, and cell death; this interaction may be one of the underlying mechanisms of HANDs (Rappaport et al., 1999; Bagashev and Sawaya, 2013; Fields et al., 2015).

Drug use is a confounding factor that contributes to neurocognitive impairment and the development of dementia (Mothobi and Brew, 2012). The common use of recreational drugs increases the incidence and severity of HANDs and other HIV-1-associated pathological disorders within HIV-1-infected individuals (Sharma et al., 2011; Hauser et al., 2012; Nair and Samikkannu, 2012).

Methamphetamine (METH) is a highly addictive and neurotoxic drug that affects the CNS (Peerzada et al., 2013) by producing reactive oxygen species (Wu et al., 2007) and dysregulating glutamate uptake in the CNS (Abdul Muneer et al., 2011). Glutamate enters dopaminergic neurons or nerve terminals and then displaces vesicular neurotransmitters in the cortex and other widespread areas of the brain (Eisch and Marshall, 1998; Davidson et al., 2001; Deng et al., 2001). In addition, METH can induce autophagy, which plays a crucial role in METH-induced neuronal death (Kongsuphol et al., 2009; Nopparat et al., 2010; Lenzi et al., 2012). HIV-Tat and METH can significantly increase apoptosis and synergistically induce autophagy in SH-SY5Y human neuroblastoma cells (Qi et al., 2011; Zeng et al., 2018). However, the molecular mechanism of HIV-Tat and METH-induced neuronal autophagy is poorly understood.

Lysosomes are digestive organelles in which double-membraned autophagosomes deliver cytoplasmic contents for degradation (Bento et al., 2016). However, extensive autophagy may be involved in type II programmed cell death or autophagic cell death, which has been linked to non-apoptotic cell death (Tsujimoto and Shimizu, 2005). The hallmark of the autophagic process is the sequestration of cytoplasmic content through the formation of double-membrane vesicles; this process is mediated by numerous evolutionarily conserved, autophagy-related (Atg) proteins (Roohbakhsh et al., 2016). Among the multitude of genetic components that are associated with the regulation of autophagy, growth, and metabolism, phosphatidylinositol-3-kinase (PI3K)/mammalian target of rapamycin (mTOR) signaling is a negative regulator of autophagy (Liang, 2010).

mTOR, the first member identified in the phosphatidylinositol kinase-related kinase family, contains two protein complexes: mTORC1 and mTORC2 (Zarogoulidis et al., 2014). mTORC1 modulates protein translation in response to environmental signals by targeting p70S6 kinase 1 and 2 and 4E-binding protein 1 (Saxton and Sabatini, 2017). mTORC2 (mTOR-rictor complex) regulates the phosphorylation and activation of Akt/PKB, protein kinase C, and glucocorticoid-induced protein kinase 1 (Sarbassov et al., 2005, 2006; Parzych and Klionsky, 2014).

The tree shrew (*Tupaia belangeri*) is a promising animal model for several human diseases in Southeast Asia because it is closely related to humans phylogenetically (Novacek, 1992). Some researchers have classified tree shrews as a separate taxonomic group of mammals (*Scandentia*) that likely diverged from the primate order (*Primates*) approximately 85 million years ago (Yang et al., 2013). As a new animal disease model, tree shrews are mainly used to study viral infections (Wu et al., 1982; Pang et al., 1984; Wang et al., 2012; Yang et al., 2013; Tsukiyama-Kohara and Kohara, 2014), neurological disorders (Feng et al., 2011; Wang et al., 2011; Ma et al., 2013), and cancers (Ye et al., 2016), as well as digestive, urinary, and metabolic diseases (Cao et al., 2003; Zhu et al., 2013; Yao, 2017).

In our study, we cultured the primary midbrain neuronal cells of tree shrews as an experimental model; then, we treated the cells with METH and HIV-Tat to examine METH and HIV-Tat-induced autophagy and study its potential mechanisms. We also studied the role of METH and HIV-Tat in mediating the protein expressions of the autophagy-related genes, including Beclin-1, LC3B, ATG5, and ATG7. In addition, we examined the effect of the inhibition of mTOR signaling via pharmacological intervention on METH and HIV-Tat-induced autophagy in primary midbrain neuronal cells. Our study facilitates the identification of the underlying molecular mechanisms of METH and HIV-Tat-induced autophagy in primary midbrain neuronal cells.

## Materials and Methods

### Animals

Tree shrews (newborns, 1–3 days old) were supplied by the Center of Tree Shrew Germplasm Resources, the Institute of Medical Biology, the Chinese Academy of Medical Science, and the Peking Union Medical College (Kunming, China). The study was approved by the Institutional Ethics Committee of Kunming Medical University and was performed in accordance with the ethical standards described in the 1964 Declaration of Helsinki and its later amendments.

### Primary Neuronal Cell Cultures and Treatments

The newborn tree shrews were sacrificed using 1% pentobarbital sodium salt (Sigma-Aldrich, United States), which was administered via intraperitoneal anesthesia. On a clean bench, the brains were removed and placed into culture dishes containing pre-cooled D-Hank’s solution. The meninges, olfactory bulb, cerebellum, and brain stem were removed to expose the midbrain. These components were harvested and washed twice with pre-cooled phosphate buffer solution (PBS). The tissues were cut into 1-mm blocks using microscissors and transferred into centrifuge tubes. Subsequently, all the samples were digested with 0.25% trypsin (Gibco, United States) for 20 min, treated with Dulbecco’s Modified Eagle Medium/High Glucose (Hyclone, United States) + 5% fetal bovine serum (Gibco, United States) to stop the digestion, and centrifuged at 2,000 g for 10 min. The dissociated cells were seeded at 5 × 10^5^ cells/well in the culture medium, which contained Neurobasal medium, B27 (1:50), 2 mM of glutamine, 1% penicillin, and streptomycin (all the solutions were obtained from Gibco, United States), and were placed in plates coated with poly-D-lysine (Thermo Fisher Scientific, United States) in 5% CO_2_ at 37°C. After 3 days, the culture medium was replaced. Seven days later, the cells were identified by immunofluorescence labeling with anti-rabbit Nestin (Abcam, UA, 1:200 with PBS) and were then treated with METH (National Institutes for Food and Drug Control, Beijing, China), HIV-Tat (Prospec, Rehovort, Israel), 3-Methyladenine (3-MA, 50 μM, Sigma-Aldrich, United States), rapamycin (Rapa, 10 nM, Sigma-Aldrich, United States), *siAtg5*, and *siAtg7*. After 24 h, the cells were harvested for subsequent experiments.

### Western Blot

The cells were treated with METH and/or HIV-Tat, 3-MA, Rapa, *siAtg5*, and *siAtg7* for 24 h, lysed in a 100 μl protein extraction buffer (mammalian cell and tissue extraction kit, Biovision, United States) containing protease and phosphatase inhibitors, and centrifuged at 13,000 g for 15 min at 4°C. The supernatant was collected for storage at −80°C for Western blot analysis. The proteins were measured using the Bradford Protein Assay kit (Beyotime, Shanghai, China), separated by 6–12% sodium dodecyl sulfate-polyacrylamide gel, and transferred onto 0.2 μm or 0.4 μm polyvinylidene difluoride membranes (Millipore, Billerica, MA, United States). The membranes were blocked at room temperature for 1 h in 5% nonfat dry milk (diluted in the Tris-buffered saline, 0.1% Tween 20, TBST) and then incubated with primary antibodies [anti-rabbit Beclin-1, anti-rabbit LC3B, anti-rabbit phosphorylated-mTOR (p-mTOR) (S2481), anti-rabbit mTOR, anti-rabbit ATG5, anti-rabbit ATG7 (these antibodies were diluted with TBST on 1:1,000 and purchased from Cell Signaling Technology, United States), and anti-mouse β-Actin (Sigma-Aldrich 1:2,000)] overnight at 4°C. Next, the membranes were washed three times for 10 min each time with TBST and then incubated with the anti-mouse/rabbit IgG and the horseradish peroxidase-linked secondary antibody (1:5,000 with 5% defatted milk, Cell Signaling Technology, United States) for 1 h at room temperature. Finally, the membranes were detected using an enhanced chemiluminescent Plus Detection kit (Millipore, United States) and visualized using a Bio-Rad Imaging system (Bio-Rad, United States). This experiment was repeated in triplicate, and representative Western blot images were presented.

### Transmission Electron Microscopy (TEM)

For TEM analysis, the cells were treated with METH and HIV-Tat for 24 h and were harvested and fixed immediately in a 2% glutaraldehyde solution buffered with PBS at 4°C overnight. The samples were sent for TEM analysis, which was conducted by the Department of Electron Microscopy in the Institute of Medical Biology, the Chinese Academy of Medical Science, and the Peking Union Medical College (Kunming, China). The sections were imaged via a TEM (H-600, Hitachi, Japan) that was operated at 80 kV.

### Fluorescence Microscopy

The cells were seeded at 1 × 10^6^ on a glass bottom dish (Cellvis, United States). After being treated with *siAtg5* and *siAtg7* for 48 h,followed by the single or combined treatment of METH and/or HIV-Tat, the cells were fixated with 4% paraformaldehyde in double-distilled water (ddH_2_O) (Solarbio, China) for 20 min and were blocked and permeabilized with 1% BSA in PBS with 0.1% Triton-100 in PBS for 30 min. Next, the cells were immunostained for anti-rabbit LC3B (Cell Signaling Technology, United States, 1:200 with PBS) overnight to analyze the autophagosomes. After being washed three times with PBS, the cells were incubated with the goat anti-rabbit antibody conjugated with Alexa Fluor 555 (Life, United States, 1:500 with 1% BSA). Finally, the nuclei were counterstained with 4′,6-diamidino-2-phenylindole (DAPI) (1:500 with PBS) and were detected using a fluorescence microscope (Olympus FluoView^TM^ 1000 Confocal microscope, Olympus, Japan). The data are summarized as the mean ± standard deviation (SD).

### Transfection With *siAtg5* and *siAtg7*

The cells were seeded on six-well plates to grow to 70% confluence, then 5 μl Lipofectamine 2000 (Invitrogen, Carlsbad, CA, United States) reagent and 30 μmol *Atg5* and *Atg7* small interfering RNA (siRNA) (*siAtg5*, *siAtg7*) (provided by Prof. LiJun Chen) were added in free serum medium for 48 h, then treated with the METH and/or HIV-Tat treatment. After being cultured for 24 h, they were harvested for subsequent experiments. The sequences of *siAtg5* and *siAtg7* were as follows: 5′-CCTGAACAGAATCATCCTTAA-3′ and 5′-CCCAGCTATTGGAACACTGTA-3′, respectively.

### Transfection With Ad-mCherry-GFP-LC3B

The cells were seeded on a glass bottom dish to grow to 80% confluence, transfected with 20 MOI of Ad-mCherry-GFP-LC3B (Adenovirus expressing mCherry-GFP-LC3B fusion protein, Beyotime, China) for 24 h, and treated with the METH and/or HIV-Tat treatment. After being cultured for 24 h, the cells were fixed with 4% paraformaldehyde and viewed using a confocal microscope, as described above.

### Statistical Analyses

Statistical analyses were performed using SPSS 19.0 (IBM SPSS, Chicago, United States) and GraphPad Prism 7.00 (GraphPad Software, United States). The Bliss Independence model was used to study the combination effects of HIV-Tat and/or METH on the Beclin-1, LC3B, ATG5, ATG7, and p-mTOR protein expressions (Foucquier and Guedj, 2015). Bliss Independence is described by the equation for probabilistic independence: E_A_ + E_B_ − E_A_ ∙E_B_, where 0 ≤ E_A_ ≤ 1, and 0 ≤ E_B_ ≤ 1. E_A_ and E_B_ are the respective effects of METH and HIV-Tat. The resulting Combination Index can be calculated as $\frac{{\text{E}}_{\text{A}}+{\text{E}}_{\text{B}}-{\text{E}}_{\text{A}}•{\text{E}}_{\text{B}}}{{\text{E}}_{\text{AB}}}$; E_AB_ is the combined effect of HIV-Tat and METH. The Combination Index is recognized as the standard measure of a combination effect to indicate a synergistic (<1), additive ( = 1), or antagonistic (>1) effect (Foucquier and Guedj, 2015). The data are summarized as the mean ± SD of three independent biological replicates. Group comparisons were performed using a one-way analysis of variance. Tukey’s *post hoc* tests were used to determine differences in the protein expression levels of Beclin-1, LC3B, ATG5, ATG7, and p-mTOR in the primary midbrain neurons of tree shrews of the different treatment groups compared to that of the respective controls. *P*-values of < 0.05 were considered statistically significant.

## Results

### METH Induces Neuronal Cell Autophagy in Tree Shrews

**FIGURE 1:**
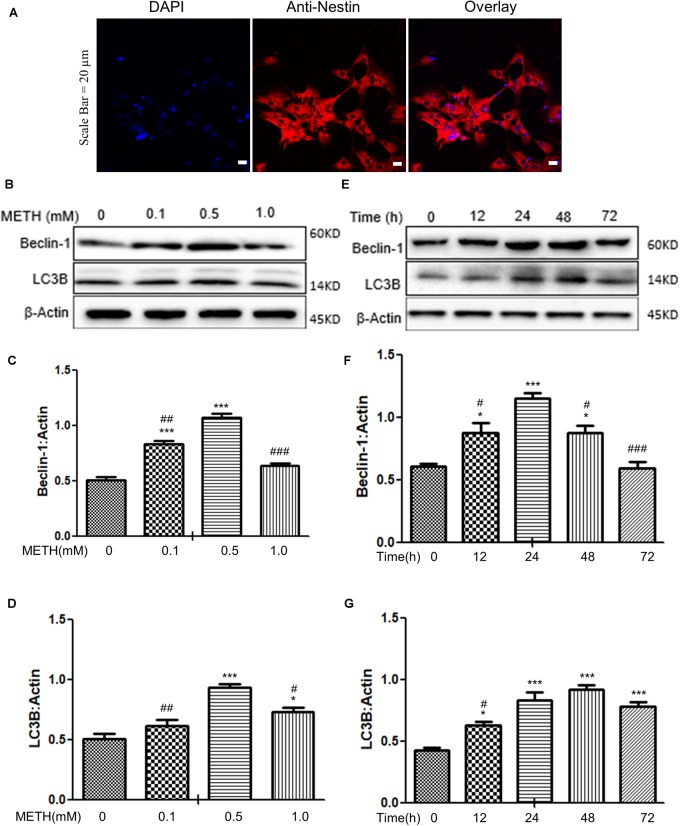
METH-induced autophagy in primary neuronal cells. **(A)** The primary cells were identified as neurons by anti-Nestin immunofluorescence. Scale bar = 20 μm. **(B–G)** The β-Actin- protein expression levels of Beclin-1 and LC3B were analyzed using Western blot and quantified by Image J software, with bars showing the means and individual data points of each column. **(B–D)** The cells were exposed to different doses (0.1, 0.5, 1 mM) of METH for 24 h. **(E–G)** The cells were exposed to 0.5 mM of METH at varying time periods (12, 24, 48, 72 h). Representative blot images were shown. ^∗^*p* ≤ 0.05, ^∗∗∗^*p* ≤ 0.001, compared to the respective controls (Ctrl = time 0 h), ^#^*p* ≤ 0.05, ^##^*p* ≤ 0.01, ^###^*p* ≤ 0.001, compared to the M (0.5 mM) and 0.5 mM METH for 24 h. The data were presented as mean ± S.D. *N* = 3 biological replicates per group.

When the primary neuronal cells of tree shrews were cultured for 7 days, the cells were identified as neurons (Figure 1A); Next, we determined the expression levels of autophagy-related protein markers (Beclin-1 and LC3B) in the METH-treated primary neuronal cells. Previous studies reported that a wide range of METH dosages from 1 μM up to 3 mM was sufficient to induce autophagy in different cell lines (Ma et al., 2014; Li et al., 2017; Zeng et al., 2018). We decided to perform a dose-dependent assay to evaluate the effects of varying METH concentrations on the expression levels of Beclin-1 and LC3B. As shown in Figures 1B–D, when the primary midbrain neuronal cells were treated with 0.1 mM of METH for 24 h, the protein expression level of Beclin-1 was elevated by 1.6 ± 0.1-fold (*p* < 0.001) compared to the saline-paired controls. When the cells were treated with 0.5 mM of METH, the expression of proteins experienced maximum increases in Beclin-1 and LC3B levels by 2.1 ± 0.1-fold (*p* < 0.001) and 1.4 ± 0.1-fold (*p* < 0.001), respectively, compared to the saline-paired controls.

In the next step, the cells were treated with 0.5 mM of METH for varying time periods. As shown in Figures 1E–G, METH significantly elevated the Beclin-1 and LC3B protein levels starting at 12 h by 1.4 ± 0.5-fold and 1.5 ± 0.4-fold (*p* < 0.05), respectively, and a maximal increase in the Beclin-1 protein level at 24 h by 1.7 ± 0.3-fold (p < 0.001) compared to the saline-paired controls; the protein expression level of LC3B peaked at 48 h by 2.7 ± 0.02-fold (*p* < 0.001), respectively, compared to the saline-paired controls. Taken together, the results showed that METH exposure increased the expression of Beclin-1 and LC3B proteins in a dose-dependent and time-dependent manner.

### METH Synergistically Induces Autophagy With HIV-Tat

**FIGURE 2:**
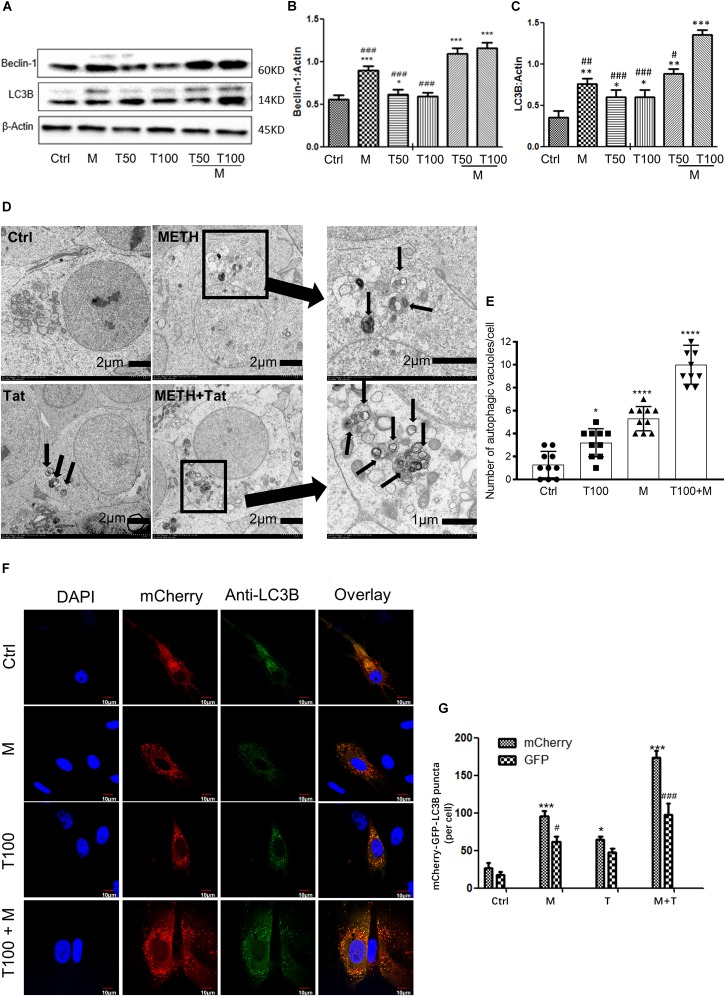
HIV-Tat synergistically induces autophagy with METH. **(A–C)** Two different doses of HIV-Tat (T50, 50 nM; T100, 100 nM) were treated in the cells alone or combined with METH (M, 0.5 mM) for 24 h. The β-Actin- protein expression levels of Beclin-1 and LC3B were analyzed using Western blot and quantified by Image J software, with bars showing the means and individual data points of each column. Representative blot images were shown. ^∗^*p* ≤ 0.05, ^∗∗^*p* ≤ 0.01, ^∗∗∗^*p* ≤ 0.001, compared to the respective controls (Ctrl). ^#^*p* ≤ 0.05, ^##^*p* ≤ 0.01, ^###^*p* ≤ 0.001, compared to the M+T100. The data were presented as mean ± S.D. *N* = 3 biological replicates per group. **(D)** Ultrastructural analysis of METH and/or HIV-Tat-treated cells by TEM. The red arrows in the micrograph represent the double membrane of the autophagic vesicles. The black boxed area contains representative autophagic vacuoles of METH and HIV-Tat-treated cells at 1,200 magnification. Arrows in the enlarged image indicate double membrane of autophagic vacuoles at 4,000/3,500 magnification. **(E)** Quantification of autophagic vacuoles per neuronal cell. A total of 10 randomly selected neurons in randomly selected fields per treatment were counted. The data were presented as mean ± S.D. ^∗^*p* ≤ 0.05, and ^∗∗∗∗^*p* ≤ 0.0001 compared to the respective controls (Ctrl). **(F)** Immunofluorescence staining of mCherry and LC3B in the METH (M, 0.5 mM) and /or HIV-Tat (T100, 100 nM)-treated primary neurons for 24 h. **(G)** Quantification of LC3B puncta per neuronal cell. A total of 10 randomly selected neurons in randomly selected fields per treatment were counted. The data were presented as mean ± S.D. ^∗^or ^#^*p* ≤ 0.05, and ^∗∗∗^ or ^###^*p* ≤ 0.001 compared to the respective controls (Ctrl-mCherry). Representative images were shown.

As METH induced the expression levels of autophagy-related protein markers (Beclin-1 and LC3B) in primary midbrain neuronal cells of tree shrews, we next examined whether METH demonstrated a combination effect with HIV-Tat in inducing the protein expressions of Beclin-1 and LC3B. To assess the role of HIV-Tat and METH-induced autophagy in primary neuronal cells, two different concentrations of HIV-Tat (50 and 100 nM) were treated individually and in combination with METH (M, 0.5 mM) for 24 h. As shown in Figures 2A–C, 50 nM HIV-Tat (T50) and 100 nM HIV-Tat (T100) did not strongly elevate the protein expression levels of Beclin-1 and LC3B. However, the combination of METH and HIV-Tat showed further increases in the protein levels of Beclin-1 and LC3B compared with METH or HIV-Tat alone (especially 100 nM METH and HIV-Tat). As shown in Figures 2A–C, 50 nM HIV-Tat could synergistically interact with 0.5 mM of METH to promote the protein expression levels of Beclin-1 and additively elevate the LC3B level with Combination Index values of 0.68 and 1.17, respectively, compared to the METH treatment alone (Please refer to the method for the combination index); 100 nM HIV-Tat was shown to further elevate the protein expression levels of Beclin-1 and LC3B with Combination Index values of 0.56 and 0.34, respectively, compared to the METH treatment alone. The results indicated that HIV-Tat synergistically interacted with METH to induce the protein expressions of the cellular autophagy markers Beclin-1 and LC3B in the primary midbrain neuronal cells of tree shrews in a dose-dependent manner.

Although the combined treatment of METH and HIV-Tat induced the expression levels of both autophagy markers, Beclin-1 and LC3B, the results did not directly refer to the introduction of autophagy. To confirm the combined effect of METH and HIV-Tat on inducing autophagy, TEM was used to observe the presence of autophagic vesicles in the cells. As shown in Figure 2D, numerous multi-membrane autophagosomes were observed in cells exposed to HIV-Tat and METH combined for 24 h, whereas few or no autophagosomes were observed in the saline-paired controls and the METH and HIV-Tat-only groups. Both the METH (0.5 mM) and HIV-Tat (100 nM) treatments significantly increased the numbers of autophagic vesicles by 4 ± 1-fold (*p* < 0.0001) and 2 ± 1-fold (*p* = 0.013); the combined treatment of METH and HIV-Tat further increased the number of autophagic vesicles by 8 ± 2-fold (*p* < 0.0001) compared to the saline-paired controls (Figure 2E).

To determine the autophagosome and autolysosome formation in response to HIV-Tat and/or METH treatment, Ad-mCherry-GFP-LC3B was used to monitor the autophagic flux, which is a coupled autophagosome formation and degradation process (Gump and Thorburn, 2014). Ad-mCherry-GFP-LC3B is a recombinant adenovirus that can effectively express the red fluorescent protein mCherry and green fluorescence in target cells after infection (Gump and Thorburn, 2014). Photoproteins (GFP) and LC3B fusion proteins present bright red and green fluorescence, which can be used to monitor autophagy. The recombinant adenovirus is a mature, E1-defective, recombinant adenovirus vector system that cannot be amplified and reorganized after infecting normal cells. After infection of cells with Ad-mCherry-GFP-LC3B adenovirus, in the case of non-autophagy, mCherry-GFP-LC3B is present in the cytoplasm in the form of diffuse yellow fluorescence (the combined effect of mCherry and GFP) under a fluorescence microscope. In the case of autophagy, mCherry-GFP-LC3B gathers on the autophagosome membrane under the fluorescence microscope and is expressed in the form of yellow spots (LC3B dot or punctae). When autophagosomes fuse with lysosomes, the parts of the GFP fluorescence quench and appear as red spots.

When the cells were induced to autophagy, Ad-mCherry-GFP-LC3B was clustered in the double membrane of the autophagosomes, to monitor autolysosome formation. As represented in Figures 2F,G, the combined treatment of HIV-Tat and METH resulted in an increase of LC3B-positive puncta compared to the saline-paired controls and the METH and HIV-Tat-only groups.

### Autophagy-Related Proteins ATG5 and ATG7 Are Involved in METH and HIV-Tat-Induced Autophagy

**FIGURE 3:**
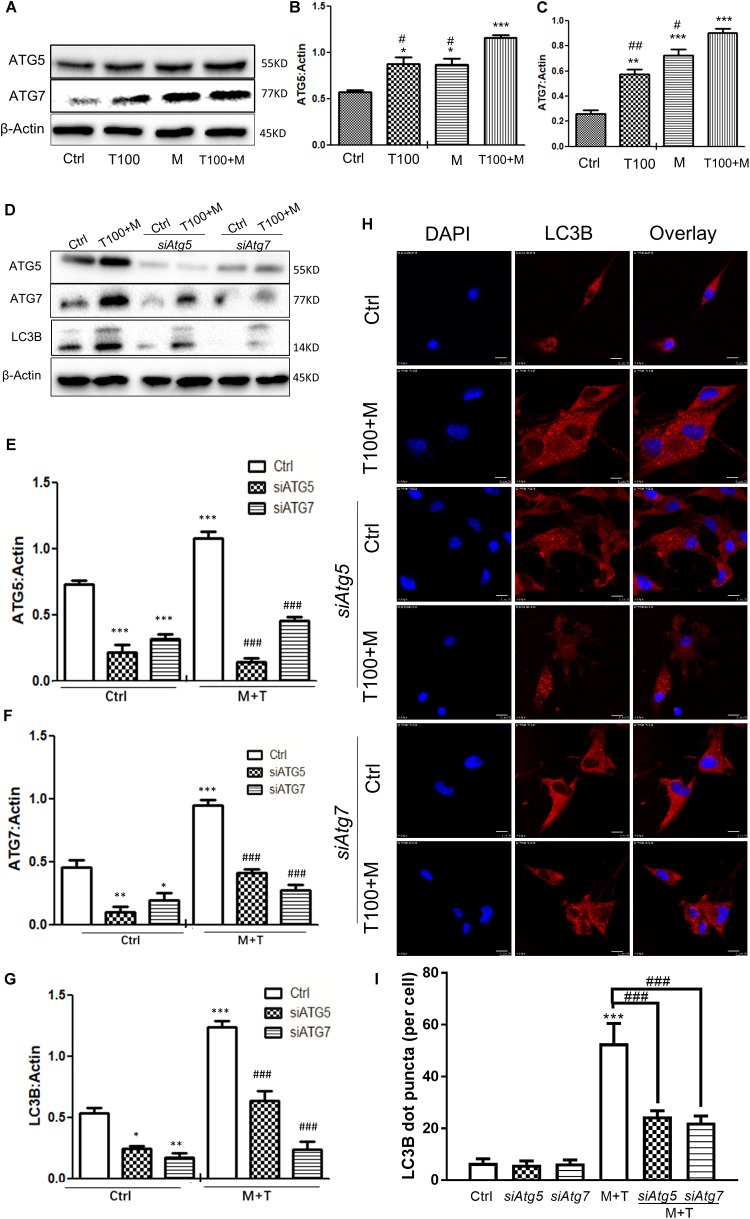
Autophagy-related ATG5 and ATG7 are involved in the METH and HIV-Tat-induced autophagy. **(A–C)** The abundance of ATG5 and ATG7 proteins were detected in HIV-Tat (T100, 100 nM) and/or METH (M, 0.5 mM)-treated cells for 24 h. The β-Actin protein expression levels of ATG5 and ATG7 were analyzed using Western blot, quantified by Image J software, and displayed using the scatter dot plot, with bars showing the means and individual data points of each column. Representative blot images were shown. ^∗^*p* ≤ 0.05, ^∗∗^*p* ≤ 0.01, ^∗∗∗^*p* ≤ 0.001, compared to the respective controls (Ctrl); ^#^*p* ≤ 0.05, ^##^*p* ≤ 0.01, compared to the M+T100. The data were presented as mean ± S.D. *N* = 3 biological replicates per group. **(D–G)** After the *Atg5* and *Atg7* genes were silenced, the protein levels of ATG5 and ATG7 were analyzed in cells with or without the HIV-Tat (T100, 100 nM) and METH (M, 0.5 mM) treatment for 24 h. Representative blot images were shown. the means and individual data points of each column. Representative blot images were shown. ^∗^*p* ≤ 0.05, ^∗∗^*p* ≤ 0.01, ^∗∗∗^*p* ≤ 0.001, compared to the respective controls (Ctrl);^###^*p* ≤ 0.001, compared to the M+T100. The data were presented as mean ± S.D. *N* = 3 biological replicates per group. **(H)** LC3B puncta were detected by fluorescence microscopy to determine the effect of ATG5 and ATG7 on the METH (M, 0.5 mM) and HIV-Tat (T100, 100 nM)-induced autophagy. Representative images were shown. **(I)** Quantification of LC3B puncta per neuronal cell. A total of five randomly selected neurons in randomly selected fields per treatment were counted. The data were presented as mean ± S.D. ^∗∗∗^*p* ≤ 0.001 compared to the control, ^###^*p* ≤ 0.001 compared to the M+T. Representative images were shown.

To determine whether the autophagy-related proteins ATG5 and ATG7 were involved in METH and HIV-Tat-induced autophagy in the primary midbrain neuronal cells of the tree shrews, the protein levels of ATG5 and ATG7 were measured. As shown in Figures 3A–C, 100 nM HIV-Tat promoted the protein expression levels of ATG5 and ATG7 by 1.3 ± 0.08-fold (*p* < 0.01) and 2.6 ± 0.4-fold (*p* < 0.01), respectively, compared to the saline-paired controls; 0.5 mM of METH was shown to elevate the protein expression levels of ATG5 and ATG7 by 1.3 ± 0.07-fold (*p* < 0.01) and 2.9 ± 0.4-fold (*p* < 0.001), respectively, compared to the saline-paired controls.

The combination of METH and HIV-Tat showed further increases in the protein levels of ATG5 and ATG7 compared with METH or HIV-Tat alone. As shown in Figures 3A–C, the combined treatment of HIV-Tat and METH increased the protein expression levels of ATG5 and ATG7, with Combination Index values of 0.60 and 0.91, respectively, compared to the METH treatment alone; this suggested a mild synergy of the combined treatment of HIV-Tat and METH with the ATG5 and ATG7 protein expressions. The results indicated the involvement of ATG5 and ATG7 in the METH and HIV-Tat-induced autophagy in the primary midbrain neuronal cells of tree shrews.

In the next step, we adopted a loss-of-function approach to further study the role of ATG5 and ATG7 in METH and HIV-Tat-induced autophagy. As shown in Figures 3D–G, the silencing of *Atg5* and *Atg7* by siRNAs strongly reduced the ATG5 and ATG7 protein expression levels by 0.8 ± 0.2-fold (*p* < 0.001) and 0.5 ± 0.5-fold (*p* < 0.05), respectively, compared to the saline-paired controls; this confirmed the potency of the siRNA constructs. *siAtg5* was shown to attenuate the ATG7 protein levels by 0.6 ± 0.05-fold (*p* < 0.001); in contrast, *siAtg7* was shown to reduce the ATG5 protein levels by 0.4 ± 0.01-fold (*p* < 0.01), respectively, compared to the saline-paired controls; this indicated the off-target effects of the siRNAs.

We demonstrated that the combined treatment of METH and HIV-Tat significantly elevated the protein expressions of ATG5, ATG7, and LC3B by 1.5 ± 0.03-fold (*p* < 0.001), 2.1 ± 0.1-fold *p* < 0.001), and 2.3 ± 0.07-fold (*p* < 0.001), respectively, compared to the saline-paired controls. However, the depletion of endogenous *Atg5* and *Atg7* caused significantly reduced the protein level of LC3B induced by the combined treatment of METH and HIV-Tat by 0.4 ± 0.03-fold (*p* < 0.001) and 0.8 ± 0.04-fold (*p* < 0.001), respectively, compared to that in the combined treatment of METH and HIV-Tat; this highlighted the potential role of ATG5 and ATG7 in METH and HIV-Tat-induced autophagy. It warrants further experiments to verify the functional specificity of ATG5 and ATG7 in METH and HIV-Tat-induced autophagy *in vivo*.

To further examine the effect of ATG5 and ATG7 on METH and HIV-Tat-induced autophagy, cells were observed by immunofluorescence staining. LC3B was used to label autophagosomes. As shown in Figures 3H,I, the METH and HIV-Tat combination increased the autophagic flux, while *siAtg5* and *siAtg7* blocked the autophagic flux induced by METH and HIV-Tat; this confirmed the functional role of ATG5 and ATG7 in the METH and HIV-Tat-induced autophagy.

### HIV-Tat and METH Induce Neuronal Cell Autophagy via the mTOR Pathway

**FIGURE 4:**
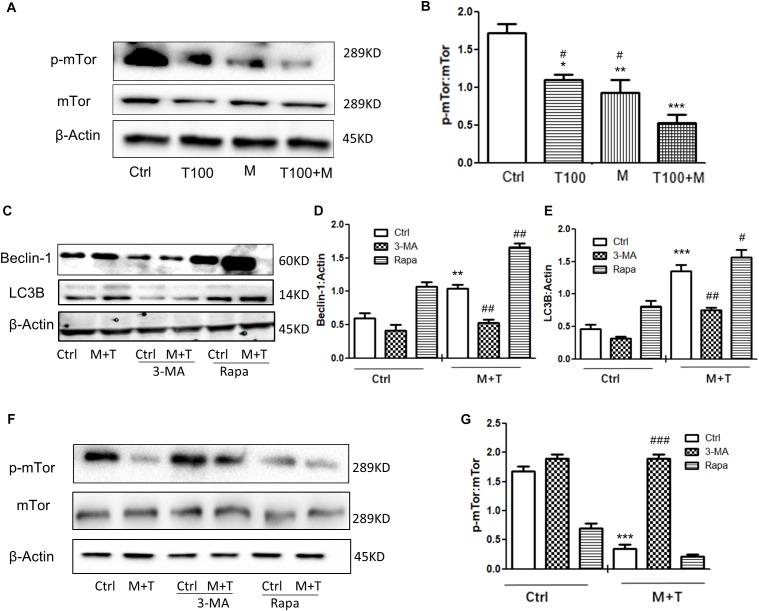
HIV-Tat and METH induce neuronal cells autophagy via the mTOR pathway. **(A,B)** The abundance of p-mTOR and non-phosphorylated form of mTOR protein were detected in the HIV-Tat (T100, 100 nM) and/or METH (M, 0.5 mM)-treated cells for 24 h. The β-Actin-normalized p-mTOR protein expression level was analyzed using Western blot and quantified by Image J software, with bars showing the means and individual data points of each column. Representative blot images were shown. ^∗^*p* ≤ 0.05, ^∗∗^*p* ≤ 0.01, ^∗∗∗^*p* ≤ 0.001, compared to the saline-paired controls (Ctrl); ^#^*p* ≤ 0.05, compared to the T100+M. The data were presented as mean ± S.D. *N* = 3 biological replicates per group. **(C–G)** After the cells were treated with or without 3-MA (50 μm) or Rapa (10 nM) and then treated with METH (M, 0.5 mM) + HIV-Tat (T100, 100 nM) or not for 24 h, the levels of Beclin-1 **(C,D)** LC3B (**C,E),** and p-mTOR **(F,G)** were detected by Western blot and quantified by Image J software, with bars showing the means and individual data points of each column. Representative blot images were shown. ^∗∗^*p* ≤ 0.01, ^∗∗∗^*p* ≤ 0.001, compared to the saline-paired controls (Ctrl); ^#^*p* ≤ 0.05, ^##^*p* ≤ 0.01, ^###^*p* ≤ 0.001, compared to the combined treatment of HIV-Tat and METH. The data were presented as mean ± S.D. *N* = 3 biological replicates per group.

mTOR is one of the upstream proteins that plays a key role in the initiation of autophagy. As shown in Figures 4A,B, when the primary midbrain neuronal cells of tree shrews were treated with the combination of METH and HIV-Tat, the phosphorylated-mTOR (p-mTOR) level was significantly down-regulated compared with the METH or HIV-Tat-only treatment and the saline-paired control group by 0.36 ± 0.01-fold (*p* < 0.05), 0.45 ± 0.02-fold (*p* < 0.01), and 0.67 ± 0.08-fold (*p* < 0.001), respectively. To determine the role of the mTOR pathway in HIV-Tat and METH-induced autophagy, the cells were treated with 3-Methyladenine (3-MA: a PI3K inhibitor) and Rapamycin (Rapa: an mTOR inhibitor) and then treated with the HIV-Tat and METH treatment for 24 h. As shown in Figures 4C–E, the Rapa pretreatment promoted the protein expression levels of Beclin-1 and LC3B by 2.1 ± 0.2-fold (*p* < 0.001) and 1.9 ± 0.1-fold (*p* < 0.001); in contrast, the 3-MA pretreatment reduced the protein expression levels of Beclin-1 and LC3B by 0.6 ± 0.1-fold (*p* < 0.05) and 0.6 ± 0.1-fold (*p* = 0.0010), respectively, compared to the saline-paired controls. Consistently, the Rapa pretreatment further elevated the protein expression levels of Beclin-1 and LC3B that were induced by the combined treatment of METH and HIV-Tat by 3.9 ± 0.2-fold (*p* < 0.001) and 2.1 ± 0.1-fold (*p* < 0.001), however, the 3-MA pretreatment further reduced the protein expression levels of Beclin-1 and LC3B by 0.4 ± 0.1 (*p* < 0.0001) and 0.6 ± 0.1-fold (*p* < 0.001) compared to the combined treatment of METH and HIV-Tat in the primary midbrain neuronal cells.

As shown in Figures 4F,G, The combined treatment of METH and HIV-Tat strongly inhibited the phosphorylation of mTOR by 0.17 ± 0.04-fold (*p* < 0.001) compared to the saline-paired controls. The 3-MA and Rapa pretreatment promoted and inhibited the phosphorylation level of mTOR by 4.6 ± 0.1-fold (*p* < 0.001) and, albeit insignificant, by 0.8 ± 0.04-fold (*p* = 0.9815), respectively, compared to the combined treatment of METH and HIV-Tat. Taken together, the results illustrated that HIV-Tat and METH induced neuronal cell autophagy via the mTOR pathway in the primary midbrain neurons of the tree shrews.

### HIV-Tat and METH Induce Neuronal Cell Apoptotic Death

**FIGURE 5:**
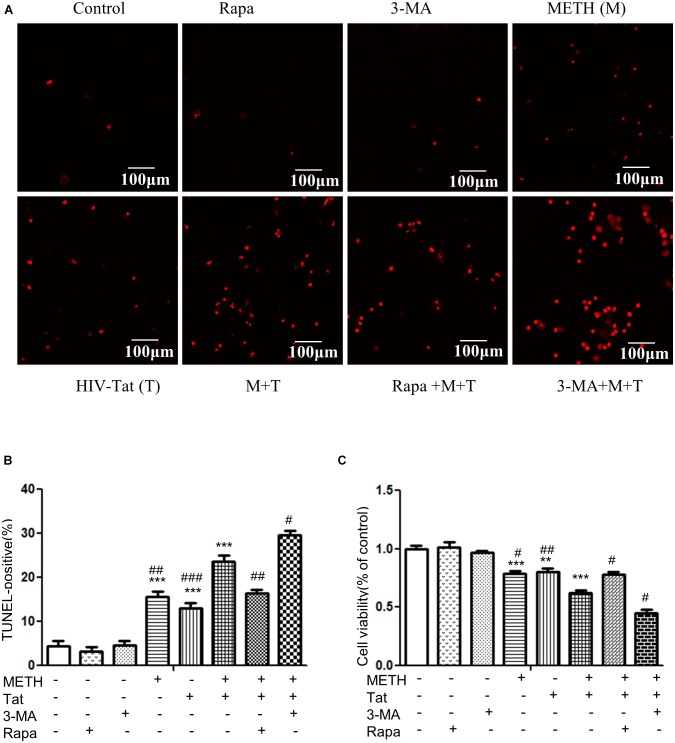
Examination of apoptosis in the METH- and/or HIV-Tat-treated neuronal midbrain cells using MTT and TUNEL assays. **(A,B)** The apoptotic cells were detected by TUNEL assay. The fragmented DNAs were labeled by after the treatment of Rapa, 3-MA and/or Meth + Tat. Scale bars: 100 μm. The percentage of TUNEL-positive cells is presented as mean ± S.E.M. ^∗∗∗^*p* ≤ 0.001compared to the control group. ^#^*p* ≤ 0.05, ^##^*p* ≤ 0.01, ^###^*p* ≤ 0.001, compared to the combined treatment of HIV-Tat and METH. A total of 10 fields were randomly selected. The data were presented as mean ± S.D. Representative images were shown. **(C)** Cells viability was detected by MTT assay. The data were presented as mean ± S.D. ^∗∗^*p* ≤ 0.01, ^∗∗∗^*p* ≤ 0.001, compared to the saline-paired controls (Ctrl); ^#^*p* ≤ 0.05, ^##^*p* ≤ 0.01, compared to the combined treatment of HIV-Tat and METH. Representative images were shown.

Autophagy is generally considered as a protective mechanism, however, extensive autophagy can cause cell death, namely type II programmed cell death (Tsujimoto and Shimizu, 2005). Thus we used a cytotoxicity assay (MTT) and Tunnel assay to determine the role of autophagy in METH- and HIV-Tat-treated midbrain neuronal cells. As shown in Figures 5A–C, the combined treatment of METH and HIV-Tat significantly reduced cell viability and increased cell death compared with the METH or HIV-Tat-only treatment and the saline-paired control, with the Combination Index value of 0.37 and 0.63, respectively, suggesting that METH and HIV-Tat have a synergistic effect on the primary midbrain neuronal cell survival. Next, we treated the cells with 3-MA and Rapa and then treated with the HIV-Tat and METH for 24 h. The results showed that 3-MA reduced cell viability and promoted cell death in METH- and HIV-Tat-treated cells, whereas Rapa increased cell viability and inhibited cell death. The results suggest that inhibition of autophagy via pharmacological intervention of the mTOR pathway could promote METH- and HIV-Tat-induced cell death, whereas enhanced autophagy could reduce cell death.

**FIGURE 6:**
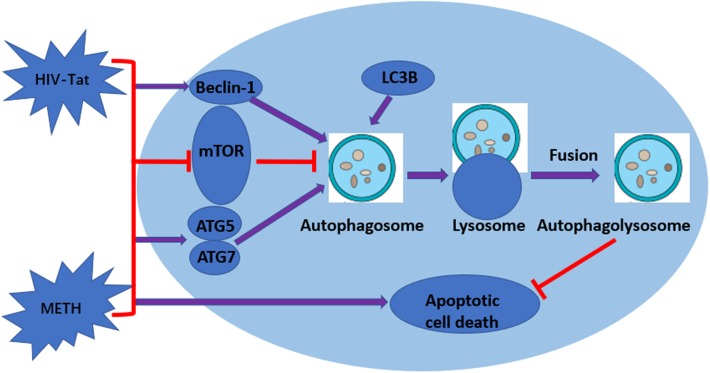
Graphical summary. METH induces autophagy among primary midbrain neuronal cells and that HIV-Tat synergistically enhances the level of autophagy.

## Discussion

Although it has been shown that HIV-Tat and METH have synergistic effects on inducing autophagy in SH-SY5Y human neuroblastoma cells, which act as dopaminergic neuronal cells (Qi et al., 2011; Zeng et al., 2018), the mechanism that underlies the induction of autophagy via HIV-Tat and METH has not been elucidated. In this study, we cultured primary midbrain neuronal cells of tree shrews and treated these cells with METH and HIV-Tat. Our results revealed that METH induced autophagy in these cells. Moreover, the effects of this induction were significantly enhanced by HIV-Tat proteins. The dosages of HIV-Tat used in this study (i.e., 50 and 100 nM) are comparable to those used in other studies to treat different cell lines. For example, three doses of HIV-Tat (6.4, 64, and 320 nM) were used to study neuronal autophagy in the B103 rat neuroblastoma cell line and E16 primary mouse hippocampal neuronal cell line (Fields et al., 2015). Additionally, the human pulmonary microvascular endothelial cells treated with 16-nM HIV-Tat were shown to enhance the expression of several autophagy markers (Dalvi et al., 2016). Previous studies indicated that HIV-Tat is secreted from infected cells and rapidly internalized by endocytosis. However, the uptake of HIV-Tat produced by infected cells, the extent to which this occurs in human CNS cells remains elusive. In HIV-infected primary Cluster of Differentiation 4 receptors (CD4^+^) T-cells, while 2/3 of the HIV-Tat produced was released, the concentration resulting from that release was 0.25 nM (Rayne et al., 2010). The serum concentration of HIV-Tat in HIV-infected individuals is approximately 0.1 nM, and from 0.1 to 0.4 nM in the culture media of HIVIIIB infected human H9 T-cells or transfected mouse T53 cells (Westendorp et al., 1995; Albini et al., 1998). The abundance of HIV-Tat in specific regions of the human CNS is yet well-defined; nonetheless, 1–3 nM HIV-Tat has been shown to potentiate or inhibit neurotransmitter release in rodent or human synaptosomes (Toborek et al., 2003; Musante et al., 2010; Zucchini et al., 2013). Though the *in vitro* neurotoxic concentration of HIV-Tat has been defined (1 nM), the *in vivo* concentration required to induce neurotoxicity is still unanswered (Eugenin et al., 2003; Agrawal et al., 2012; Bertrand et al., 2013). Considering that the concentration and distribution of HIV-Tat in different brain compartments are not well characterized, the extent to which HIV-Tat induce neurotoxicity is unclear. On the other note, HIV-infected METH users were shown to exhibit higher plasma viral loads (Moore et al., 2012; Feldman et al., 2015). However, the association between the METH and HIV-Tat in circulation/CNS is very limited. METH, in the concentrations of between 1 μM and 3 mM was shown to induce *in vitro* (Cadet et al., 2009; Kosten et al., 2014; Cao et al., 2016).

Our results also indicated that ATG5 and ATG7 were required to regulate the protein expression of LC3B and the autophagic flux induced by METH and HIV-Tat in the regulation of METH and HIV-Tat-induced autophagy, which was demonstrated by the silencing of the *Atg5* and *Atg7* genes in the primary midbrain neuronal cells. Our findings further demonstrated that METH and HIV-Tat triggered the autophagy of primary midbrain neuronal cells by inhibiting the phosphorylation of mTOR.

Autophagy is an important process for preserving cell homeostasis, which is considered a pro-survival mechanism (Ryter et al., 2013). However, autophagy also leads to cytotoxicity and cell death when the process exceeds a crucial threshold (Marino et al., 2014). A series of autophagy-related genes are involved in autophagy regulation. Beclin-1 and LC3B are the key proteins for the normal function of autophagy (Wang et al., 2013). LC3B is now widely used to monitor autophagy, as the amount of LC3B localized on the surface of autophagosomes is a reliable marker of autophagosomes (Yoshii and Mizushima, 2017). Therefore, comparing the abundance of LC3B levels between different samples is considered a reasonable method to monitor autophagic activity (Mizushima and Yoshimori, 2007). In our report, Beclin-1 and LC3B were used to monitor the level of METH and HIV-Tat-induced autophagy. The results showed that METH increased the protein expression levels of Beclin-1 and LC3B in a dose-dependent and time-dependent manner. HIV-Tat combined with METH significantly increased the levels of Beclin-1 and LC3B in primary midbrain neuronal cells (Figure 6).

Forty ATGs have been identified, 15 core members that are encoded in yeast are found to be evolutionarily conserved in mammalian systems (Shibutani et al., 2015). Several ATG complexes, the PI3K complex, and two ubiquitin-like conjugation systems are required for autophagosome formation (Mizushima and Komatsu, 2011). ATG5 conjugates ATG12 to form the ATG12-ATG5 conjugate, and ATG7, as a unique E1 enzyme, activates ATG12 and transfers it to different E2 enzymes (Noda et al., 2013). Therefore, ATG5 and ATG7 are essential for autophagy. Previous researchers have found that ATG5 and ATG7-dependent autophagy play a critical role in morphine addiction, as well as in neuronal dendritic regulation (Su et al., 2017). In our study, the combination of METH and HIV-Tat significantly up-regulated the levels of ATG5 and ATG7. Furthermore, when the *Atg5* and *Atg7* genes in the primary cells were silenced, the expression of LC3B induced by METH and HIV-Tat was reduced. This indicated that ATG5 and ATG7 were involved in METH and HIV-Tat-induced autophagy. Interestingly, we found that silencing the *Atg5* gene down-regulated the level of ATG7, whereas silencing the *Atg7* gene down-regulated the level of ATG5 in primary midbrain neuronal cells; this revealed that ATG5 and ATG7 mutually regulated each other. However, further studies are needed to elucidate the exact mechanism by which ATG5 and ATG7 regulate METH and HIV-Tat-induced autophagy.

mTOR, a crucial regulator of cellular metabolism, plays a pivotal role in the regulation of autophagy (Kim and Guan, 2015). A widely accepted upstream regulator of mTOR is the PI3K/AKT signaling pathway (Kim and Guan, 2015). In this study, we showed that METH and/or HIV-Tat down-regulated the level of phosphorylated-mTOR (p-mTOR). To further test the involvement of the mTOR-dependent pathway in the process of the METH and HIV-Tat-induced autophagy, we applied Rapa, a lipophilic macrolide antibiotic, which is a specific inhibitor of mTOR (Barber and Ganti, 2011), to primary midbrain neuronal cells before treatment with METH and HIV-Tat. The levels of p-mTOR decreased, whereas the levels of LC3B and Beclin-1 increased. We also applied 3-MA, a specific inhibitor of PI3K, to the cells. The results indicated that 3-MA elevated the protein expression of p-mTOR compared with no-3-MA groups. The combined treatment of METH and HIV-Tat significantly inhibited the phosphorylation of mTOR compared to the saline-paired controls. Combining these results, we concluded that inhibiting PI3K via pharmacological intervention induced a reduction in autophagy and promoted the expression of p-mTOR proteins and that the mTOR-dependent pathway was involved in the METH- and HIV-Tat-induced autophagy in primary midbrain neuronal cells (Figure 6). It warrants further investigation into whether and how METH and HIV-Tat regulate the translational output of the autophagy-associated Beclin-1 and LC3B at the transcriptional, post-transcriptional or post-translational level via the mTOR-mediated signaling pathway *in vitro* and *in vivo*.

Many studies have demonstrated that METH exacerbates HIV-Tat-induced neurotoxicity more than either METH or HIV-Tat alone (Mediouni et al., 2015). Prior results have shown that both METH and HIV-Tat induce the autophagy of neuronal cells (Kanthasamy et al., 2006; Li et al., 2012; Bruno et al., 2014; Fields et al., 2015). Our results confirmed that METH and HIV-Tat have a synergistic effect on inducing autophagy in primary midbrain neuronal cells. However, the effect of autophagy remains unclear. Cao et al. (2016) found that autophagy prevented cells from undergoing cell death in response to the stress induced by METH and HIV-gp120. In contrast to their findings, other researchers have suggested that METH interrupts autophagy before the lysosomal protein degradation phase (Roohbakhsh et al., 2016). In our study, we found that autophagy induced by METH and HIV-Tat exhibits a cytoprotective effect to reduce cell death. However, further studies are needed to elucidate the effects of METH and HIV-Tat-induced autophagy on neurotoxicity.

## Conclusion

Taken together, our data indicate that METH induces autophagy among primary midbrain neuronal cells and that HIV-Tat enhances the level of autophagy. ATG5, ATG7, and mTOR are involved in the autophagic signaling pathway. In addition, our data showed a clear synergistic effect of METH and HIV-Tat on neurotoxicity in primary midbrain neuronal cells. The inhibition of mTOR via pharmacological intervention strongly promotes METH and HIV-Tat-induced autophagy. The METH- and HIV-Tat- induced autophagy provides a cytoprotective effect to reduce cell death. The results were graphically summarized in Figure 6. Our findings will facilitate the identification of the underlying molecular mechanisms of the METH and HIV-Tat-induced autophagy and provide directions for future research.

## Author Contributions

JL, WW, PT, CK-L, GY, ZL, NL, XS, YH, CL and DK participated in literature search, experimental validation, data mining, bioinformatics analysis and interpretation, writing the manuscript, creating figures and tables. JD and XZ supervised the study design, critically read and edited the manuscript. All authors approved the final version for publication.

## Conflict of Interest Statement

The authors declare that the research was conducted in the absence of any commercial or financial relationships that could be construed as a potential conflict of interest.
